# Recovery, Assessment, and Molecular Characterization of Minor Olive Genotypes in Tunisia

**DOI:** 10.3390/plants9030382

**Published:** 2020-03-20

**Authors:** Olfa Saddoud Debbabi, Monica Marilena Miazzi, Olfa Elloumi, Mahdi Fendri Fendri, Fathi Ben Amar, Michele Savoia, Sara Sion, Hana Souabni, Sameh Rahmani Mnasri, Selma Ben Abdelaali, Fadwa Jendoubi, Giacomo Mangini, Franco Famiani, Francesca Taranto, Cinzia Montemurro, Monji Msallem

**Affiliations:** 1 Banque Nationale de Gènes, Boulevard du Leader Yesser Arafet, Charguia 1, 1080 Tunis, Tunisie; mnasrisameh@yahoo.fr (S.R.M.); selmabenabdelaali@gmail.com (S.B.A.); 2 Olive Tree Institute, Station régionale de Tunis, Avenue de l’Indépendance, 2049 Ariana, BP 208 Cité Mahrajène, 2049 Tunis, Tunisie; fendrimahdi@yahoo.fr (M.F.F.); msallemonji@yahoo.fr (M.M.); 3 Department of Soil, Plant and Food Sciences (DISPA), University of Bari, Via Amendola 165/A, 70126 Bari, Italy; micsav87@gmail.com (M.S.); sarasion86@gmail.com (S.S.); giacomo.mangini@uniba.it (G.M.); cinzia.montemurro@uniba.it (C.M.); 4 Olive Tree Institut Route de l’Aéroport, km 1,5 - BP 1087, 3000 Sfax, Tunisie; olfa.elloumi@iresa.agrinet.tn (O.E.); fathibenamar@yahoo.fr (F.B.A.); souabni.hana@gmail.com (H.S.); fadwa.jendoubi@hotmail.com (F.J.); 5 Department of Agricultural, Food and Environmental Sciences, University of Perugia, 06123 Perugia, Italy; franco.famiani@unipg.it; 6 CNR-IBBR Institute of Bioscience and BioResources, Via Universita’ 133, 80055 Portici, Naples, Italy; francesca.taranto@uniba.it; 7 National Research Council of Italy (CNR), Institute for Sustainable Plant Protection – Support Unit Bari, Via Amendola 122/D, 70126 Bari, Italy

**Keywords:** olive germplasm, molecular polymorphism, SSR, Tunisia

## Abstract

Olive is one of the oldest cultivated species in the Mediterranean Basin, including Tunisia, where it has a wide diversity, with more than 200 cultivars, of both wild and feral forms. Many minor cultivars are still present in marginal areas of Tunisia, where they are maintained by farmers in small local groves, but they are poorly characterized and evaluated. In order to recover this neglected germplasm, surveys were conducted in different areas, and 31 genotypes were collected, molecularly characterized with 12 nuclear microsatellite (simple sequence repeat (SSR)) markers, and compared with 26 reference cultivars present in the Tunisian National Olive collection. The analysis revealed an overall high genetic diversity of this olive’s germplasm, but also discovered the presence of synonymies and homonymies among the commercialized varieties. The structure analysis showed the presence of different gene pools in the analyzed germplasm. In particular, the marginal germplasm from Ras Jbal and Azmour is characterized by gene pools not present in commercial (Nurseries) varieties, pointing out the very narrow genetic base of the commercialized olive material in Tunisia, and the need to broaden it to avoid the risk of genetic erosion of this species in this country.

## 1. Introduction

Olive (*Olea europaea* var. *sativa* Hoffm. and Lk.), with 715 million olive trees covering an area of more than 7 million hectares, is one of the most important fruit trees in all the countries overlooking the Mediterranean Sea [1]. Olive is a multifunctional, long-living tree crop, important not only for olive and oil production, but also for characterizing, shaping, and protecting the landscape [2]. It is considered a symbol of the Mediterranean cultural heritage, an emblem of longevity and unity since the time of Roman domination, when it constituted a political and religious myth [3]. In Tunisia, its history dates back to the Phoenicians and Romans, whose commercial exchanges contributed to developing the gene fluxes and promoted the introgression of alleles from oleaster and other *O. europaea* subspecies, allowing the olive germplasm to be continuously diversified [4,5,6].

Tunisia is the fourth largest olive oil producing country in the Mediterranean Basin, owing to its 82 million olive trees covering an area of 1.84 million hectares [7]. Olive oil represents 40% of the overall value of agronomic exports of the country, and, as a primary source of income for the Tunisian people, it is a main factor of economic and social stability. Furthermore, this species, adapted to the severely hot climatic conditions, plays an important ecological role in the preservation of the environment and in the fight against desertification. 

Tunisia accounts for about 200 cultivars and genotypes, but only 58 are registered in the official national register, based mainly on pomological and morphological traits [8] or oil quality [9]. Ninety percent of the national olive oil production derives from only two highly productive varieties: ‘Chemlali’ in the central-southern region and ‘Chetoui’ in the northern region [10]. Many minor cultivars are still present in marginal areas of the country, and maintained by farmers in small local groves, but they are largely underknown. These secondary olive varieties could represent an important source of genes with a great potential for improving oil quality and introducing labeling for typical oils [11]. For these reasons, interest in this germplasm is growing, and the initiatives for its conservation and enhancement are multiplying.

Another major issue in Tunisian olive production is the lack of a varietal certification system for the propagation material, this results in frequent problems related to the varietal identification of commercialized plant material in the presence of varietal clones [12]. 

In this framework, gaining knowledge about olive genetic diversity could help tighten up the authentication of Tunisian germplasm and the implementation of new breeding programs. The studies conducted so far were mainly focused on a few economically important varieties [13,14,15,16,17,18], while the rapid development of the olive growing sector pushes us to establish a national databank for the entire olive germplasm present in Tunisia. To address these needs, an international project (Tunisian plant genetic resources better conserved and valued), coordinated by CIHEAM-Bari, was funded by Cooperazione Italiana to support the Banque des Genes Tunisienne and other public scientific Tunisian institutions. The aim of the project was to efficiently recover new germplasm in the territory, and to genetically characterize it. Simple sequence repeat (SSR) markers were chosen, as they are still considered highly reliable in the identification of varieties of different crops [19,20,21,22], including olive [23,24,25,26], population genetics [4,5,6], and product traceability [27,28,29,30]. 

This paper reports the results of the project, addressing the following aspects: i) the recovery of new germplasm from marginal areas; ii) the genetic identification of this germplasm, solving cases of homonyms and synonyms; iii) the definition of an allelic consensus list; iv) the improvement of knowledge about the genetic variability of Tunisian germplasm; v) the enrichment of the reference collection of Tunisian olive varieties.

## 2. Results

### 2.1. Genetic Diversity of Olive Genotypes 

The SSR analysis produced a total of 124 alleles, ranging from the minimum of 4 at locus DCA15 to 19 at locus DCA16 (mean 10.33 alleles/locus) (Table 1; Table S1). Values of the Shannon information index (I) ranged from 0.88 for locus DCA15 to 2.51 for DCA16 (mean 1.76). A wide genetic variation was observed, as indicated by the high values of observed (Ho) and expected (He) heterozygosity. Ho ranged from 0.25 for DCA17, to 0.97 for both DCA16 and GAPU101 (0.73 in average); He ranged between 0.45 (DCA15) to 0.89 (DCA16) (average 0.76). The mean observed heterozygosity was slightly lower than the mean expected heterozygosity, determining a positive fixation index (F) at 5 loci (mean F = 0.05) (Table 1). 

The value of the total probability of identity for the 12 SSRs analyzed, which indicates the probability that two unrelated genotypes chosen at random from all genotypes have the same profile, was very low (3.9 × 10^−15^) (Figure S1). This result suggests that the identical profiles are synonyms. 

The estimation of pairwise relatedness revealed three cases of synonymy at the Lynch and Ritland estimator LRM value of 0.50 (i.e., strong relationship between two samples): GERBOUI1/RKHAMI; MESKI2/NIB2/BESBESSI2/BESBESSI3/UNKNOWN1; ZALMATI/CHEMLALI_SFAX2 (Table S2). These identities at all the SSRs considered, confirmed also by the identity analysis conducted with Cervus, include samples all originating from the region of Ras Jibal. Identity was found also for ZALMATI/CHEMLALI_SFAX2 from the the Reference (IO) collection. The LRM cut-off at 0.35 highlighted a dense network of close relationships between many other genotypes, such as BAROUNI and BESBESSI1 from the IO collection: OCTOUBRI and RAJOU3; UNKNOWN4 and BESBESSI2/NIB2/MESKI2; and UNKNOWN2 with CHEMLALI_AZ (Table S2).

Among the 77 genotypes, seventeen showed private alleles (Table S3), with the highest number displayed by genotype TAMRI DOUIRET from the nurseries collection (4 alleles) and SAYALI3 from the IO collection (3 alleles)

To make some additional observation on the different collections, an AMOVA analysis was performed, assigning 89% of the molecular variance to differences within groups and 11% among the four groups (Table S4). Thus, the diversity indices were calculated for each of the four sampling groups of genotypes having different origin (Table 2, Figure S2). The reference group (IO collection) was the richest in alleles, with a total of 92, followed by the AZ group, with 82 alleles, the NS group, with 77 alleles, and the RJ group, with 74 alleles. While the mean expected heterozygosity was similar in the four groups, Ho was higher in the Azmour and Raz Jbal collections, resulting in a negative F for both these groups. Regarding the private alleles in the different groups, the highest number was found in the GRgroup (18 alleles), while the Ras Jbal group had the lowest (2 alleles) (Table S3). 

### 2.2. Genetic Relationships Among Olive Genotypes

**Figure 1:**
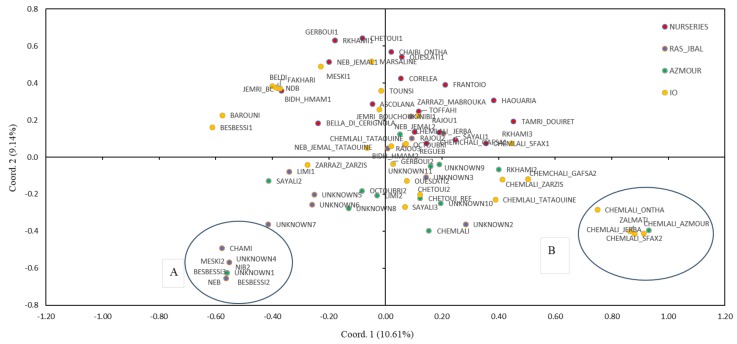
Principal coordinate analysis of 77 Tunisian olive genotypes based on their SSR polymorphism revealed by 12 SSR markers. The olive genotypes assigned to the four sampling groups are marked with colored symbols.

The genetic relationships of the Tunisian olive cultivars and genotypes were highlighted in the principal coordinate Analysis, based on Nei’s unbiased genetic distance matrix (Figure 1). The first (PCo1) and second (PCo2) principal coordinates explained a very low fraction of the variation in the molecular data, 10.61% and the 9.14%, respectively. In particular, the PCo2 discriminated most of the Ras Jbal genotypes from the IO and nurseries collections. The 26 reference varieties (IO) were intermixed with the commercial varieties (“nurseries” collection) on the two uppermost quadrants, while the Azmour and Ras Jbal samples, including all the unknown samples, clustered in the lowermost quadrants, with several genotypes forming two small clusters far from most of the samples. Cluster A groups the genotypes from Ras Jbal (UNKNOWN1, UNKNOWN4, BESBESSI2, BESBESSI3, CHAMI, MESKI2, NEB, and NIB2). Cluster B includes four Chemlali genotypes (ONTHA, AZMOUR, JERBA, and SFAX2) and the genotype ZALMATI; interestingly, several other Chemlali samples, (TATAOUINE, SFAX1, JERBA, GAFSA2, and ZARZIS) are well scattered and far away one from another, suggesting a great genetic variation of these genotypes. 

**Figure 2:**
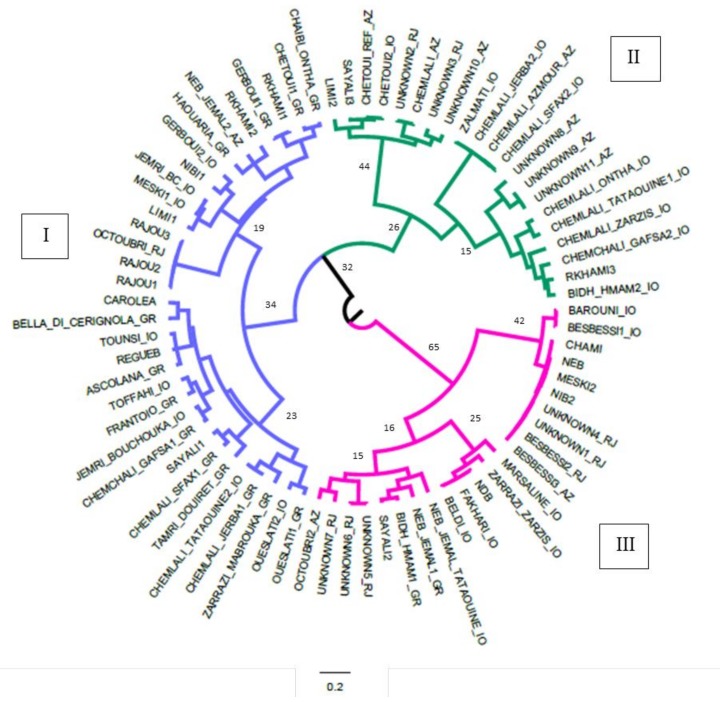
Dendrogram resulting from Ward’s hierarchical cluster analysis for 77 Tunisian olive genotypes based on 12 SSR markers, obtained with DARWIN v. 6.0.010. IO (Istitut de l’Olivier); GR (nurseries); RJ (Ras Jbal); Az (Azmour).

In order to confirm the results, a cluster analysis was carried out based on Ward’s method to maximize the between-cluster variance. The obtained dendrogram is shown in Figure 2. Genotypes were grouped in three main clusters. Cluster I included 85% of GR samples, 33% of RJ plants, 30% of IO genotypes, and two Azmour plants, NEB_JEMAL2_AZ and RKHAMI2_AZ. Cluster II included 61% of AZ genotypes, 42% of IO samples, and two RJ nnknown genotypes. Interestingly, this group included eight Chemlali genotypes (CHEMLALI_AZ, JERBA 2_IO, AZMOUR_AZ, SFAX2_IO, ONTHA_IO, TATAOUINE1_IO, ZARZIS_IO, and GAFSA2_IO), while the other four Chemlali variants collected in southern Tunisia (SFAX1_GR, JERBA1_GR, TATAOUINE 2_IO, and GAFSA1_GR) were included in Cluster I. Cluster III included all Ras Jibal “unknown” samples, except UNKNOWN2_RJ and UNKNOWN3_RJ, which belonged to Cluster II. 

### 2.3. Genetic Structure 

**Figure 3:**
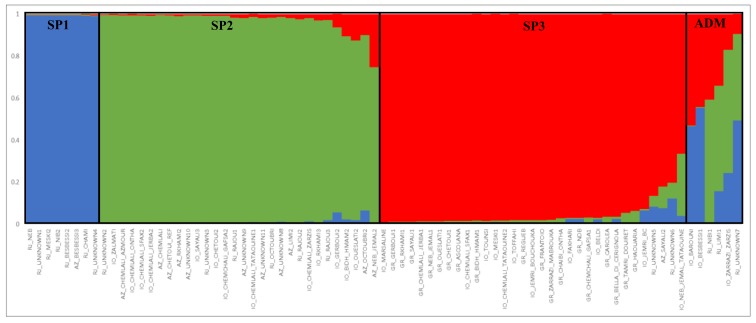
The genetic structure of 77 olive genotypes identified by the STRUCTURE algorithm at K = 3. IO: Istitut de l’Olivier; GR: nurseries; RJ: Ras Jbal; Az: Azmour; SP1: subpopulation 1; SP2: subpopulation 2; SP3: subpopulation 3; Adm: includes samples not assigned to a single subpopulation.

Application of the Bayesian clustering model implemented in STRUCTURE software with genotyping data generated by 12 SSR markers, yielded K = 3 as the best number of subpopulations (SP) for the data (Figure S3). Thus, the olive collection showed a genetic structure split into three subpopulations and a few admixed genotypes (Figure 3). The results indicated that most of the samples had a high membership in their own cluster (>97%). SP1 included only eight samples collected in the Ras Jbal group; SP 2 included eight of the Chemlali variants present in the four sampling groups, and other cultivars known as good producers of oil, such as Zalmati and Chetoui from the IO collection. SP 3 included three Chemlali genotypes and other genotypes derived from the nurseries and IO reference collections. 

An F_ST_ analysis was conducted on the three groups obtained in STRUCTURE; the results indicated great genetic differentiation between group SP1 and both SP2 (F_ST_ = 0.21) and SP3 (F_ST_ = 0.21) (Table S5). 

**Figure 4:**
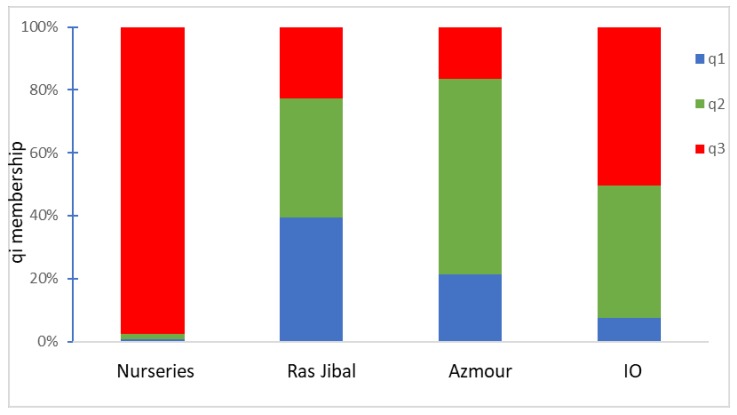
The stacked bar plots show the estimated membership coefficient (qi) relative to the subpopulations (SP1, SP2, SP3) identified by STRUCTURE for K = 3 for olive populations originating from different geographical areas.

To better understand the structure of the collection, it was divided into four a priori defined groups based on the sampling area of the collections. In each group, the mean q determined by structure analysis was calculated, resulting in a different stratification of the population (Figure 4). In particular, one main subpopulation (q3 in red) was present in the “nurseries” collection, while two different main genetic components (q2 in orange and q1 in blue) were present in the Ras Jbal, Azmour, and IO olive collections, but they were rare (<5%) in the “nurseries” collection (Figure 4). 

## 3. Discussion 

Olive is a very important crop in Tunisia, which is the fourth biggest producer in the Mediterranean area, generating around 800,000 olive tons/year, mostly from two highly productive varieties: Chemlali and Chetoui [8]. Despite this, the territory still holds a large genetic diversity for the species [31], both for cultivated and feral forms that are localized in remote areas of the country. For these neglected varieties there is, in many cases, poor information about the identity, name, and characteristics, being often guaranteed only by the personal memory of farmers. These marginal genotypes, well adapted to the extreme environmental conditions typical of the country, could have a great potential for olive genetic breeding, holding characteristics that could help in improving the long-term productivity and enhancing the competitiveness of the sector in a globalized market, especially in marginal agricultural areas. Today, there is a strong interest in the recovery and preservation of agro-biodiversity, and several projects are in place to avoid the loss of this patrimony, setting up recovery collections [32,33,34,35]. In Tunisia, although there are institutions, such as the Olive Institute, that hold a large number of olive genotypes, it is still necessary to enlarge the existing collection and develop new conservation management strategies [36,37,38]. At the same time, it is crucial to improve the plant material certification circuit to be more competitive in the global market, offering plant material with high quality standards [39]. 

To achieve these goals, in 2018, the Tunisian Gene Bank and other public scientific Tunisian institutions carried out a project to collect marginal olive germplasm throughout the country and from plant material commercialized by nurseries, performing the molecular fingerprint of these genotypes to characterize/identify them through comparison with the reference cultivars available at the IO collection. The evaluation of the samples was based on a panel of SSR markers used at the international level [40,41]. All SSR loci showed a high polymorphic information content, confirming the informativeness of these markers related to their multiallelism [42,43], and their usefulness in distinguishing the genotypes. The genetic analysis revealed high allele richness, heterozygosity, and Shannon index values at the loci analyzed, highlighting the high genetic diversity of Tunisian olive’s germplasm, as has been found for other Mediterranean countries [44,45,46,47]. 

The genetic indices calculated within each single group, IO, nurseries, Ras Jbal, and Azmour, allowed additional considerations to be made about the composition of the Tunisian germplasm. Indeed, the IO reference collection appeared to be the richest in alleles, together with that of the “nurseries” collection, which includes the “foreign” cultivars such as the Italian varieties Ascolana, Bella di Cerignola, and Carolea that are commonly commercialized in Tunisia. The two reference and commercial collections displayed most of the private alleles, in particular, genotype Tamri Douiret, from the GR collection, and Sayali3, from the IO collection, with four and three private alleles, respectively. Interestingly, several private alleles were also present in the natural “Azmour” collection. This result points out a peculiarity of the marginal “Azmour” germplasm from the perspective of a search for new and beneficial alleles; this could be important for facing incoming needs (i.e., fruit-bearing, vegetative and reproductive growth responses, resistance traits, etc.). 

The genetic relationships of the Tunisian olive cultivars and genotypes were highlighted in the PCo analysis, where several samples from the Ras Jbal collection formed a group far from the rest of the genotypes, underlining its genetic distance from the rest of the germplasm. In addition, several Chemlali samples (TATAOUINE, SFAX1, JERBA, GAFSA2, and ZARZIS) appeared well scattered and far away one from another, suggesting they are phenotypically similar but genetically different. This result points out the problem of the clonal variants in the Tunisian olive germplasm, notably for the Chemlali variety. In Tunisia, Chemlali is a generic name to indicate a genotype with a good oil production and small fruits. It is probable that this variety has many genetic variants specific to different geographical regions (Tataouine, Sfax, etc.) that can be confused during the intense exchanges of germplasm. Structure analysis clustered most of the Chemlali variants together with other good oil producer cultivars, such as Chetoui and Zalmati, with which it is often mistaken due to the high similarity for morphological and chemical characters [48], confirming that they share a common gene pool. It will be interesting, in further work, to verify the possibility of identifying characters/genes that influence the components of olive oil production through the comparative analysis of morphological and genetic traits. 

The cluster analysis, coupled with the LRM analysis confirmed the presence in the Tunisian germplasm of several synonymies and misnaming cases, such as that between genotypes GERBOUI1/RKHAMI; MESKI2/NIB2/BESBESSI2/BESBESSI3/UNKNOWN1; and ZALMATI/CHEMLALI_SFAX2. These samples all originate from the region of Ras Jibal, and it is probable that these synonymies are due to erroneous appellation of a single genotype in this marginal area. Out of 10 unknown genotypes, only UNKNOWN1_RJ was identified as a BESBESSI; the other unknown genotypes were found to be similar but not perfectly matched to known varieties; thus, they are worth investigating further to see if they can be considered as new varieties.

The cluster analysis indicated a strong relationship between the Tunisian reference varieties of the National Olive Institute of Sfax with those mainly marketed in Tunisia (nurseries). This evidence confirms the genetic correspondence among the commercial material and the reference varieties, including the Italian varieties widely spread in Tunisia. These results will be very useful to start the plant material certification process in Tunisia, following the procedures commonly adopted [38]. On the contrary, the germplasm recovered from Azmour and Ras Jbal clustered separately, indicating a differentiation from the National germplasm, and underlying the presence of an original genetic component never investigated before. This was also confirmed by the observation of the mean qi within the four a priori defined groups based on geographical origin. Only one gene pool was assigned to the “nurseries” collection, while two other gene pools were present in the Ras Jbal and Azmour olive collections. This narrow genetic basis of the “nurseries” genotypes underlines the concrete risk of genetic erosion in a crop such as the olive, which is not particularly subject to plant selection programs. On the contrary, Ras Jbal and Azmour germplasm showed a large genetic diversity totally absent in the varieties sold by “nurseries”, indicating that these sites preserve an unexplored genetic background that could be useful for investigation in a deeper way. In addition, the limited presence of this pool in the IO subpopulation confirms the validity of the recovery actions implemented by this research. 

Plant genetic resources will be essential to adapting crops to the effects of climate changes; their recovery and valorization are a first step towards the enhancement of the Tunisian olive genetic resources, which have proven to be rich and worthy of preservation. Our results indicate the need to better characterize the Tunisian germplasm in the different areas of the country, emphasizing the crucial need to proceed with the realization of a national varietal certification system for the Tunisian olive germplasm to guarantee the genetic authenticity for the commercial varieties. The usefulness of SSRs was once again confirmed in the genotyping of the Tunisian germplasm, providing highly informative data for multilocus discrimination of individuals, and shedding light on their composition and structure. In the future, the Tunisian germplasm could be better explored with innovative techniques, coupling the use of SSRs with the more performant, high throughput technologies that use next generation sequencing [49,50,51,52]. These will help to bring out the richness of the Tunisian olive germplasm, improving its commercial value. 

## 4. Materials and Methods 

### 4.1. Plant Material 

**Figure 5:**
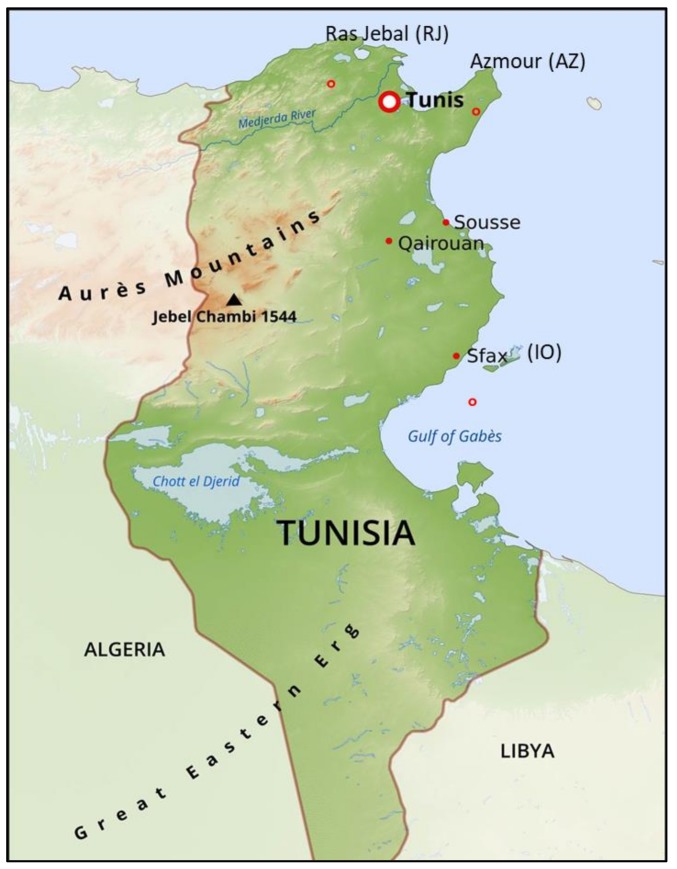
Geographical origin of the olive genotypes considered in this study.

Surveys were conducted on Tunisian farms in cooperation with local agricultural authorities and international olive experts in northern, central, and southern regions of Tunisia (Table S6, Figure 5). Seventy seven samples were collected, including 31 marginal genotypes growing at the sites of Ras Jbal (37°12′54”N, 10°07′26”E) at Bizerte governorate, and Azmour (36°55′28”N, 11°00′25”E) in the Cap Bon region; 20 commercial varieties representing the main cultivars marketed in Tunisia, obtained from commercial nurseries (tagged, nurseries collection); and 26 national varieties used as references, obtained from the National Olive Institute (34°56′08”N, 10°36′54”E, Sfax) (tagged as IO). 

### 4.2. DNA Extraction

Three young leaves of each olive sample were lyophilized and finely ground; 50 mg of tissue was used for genomic DNA extraction following the protocol described in Spadoni et al. [53]. In order to verify DNA quality and concentration, 1% agarose gel and a Nano Drop TM ND2000c (Thermo Scientific, MA, USA) spectrophotometer were used. DNA was transferred into 96-well plates and normalized to a standard concentration of 50 ng/µl and stored at -20 °C until used.

### 4.3. SSR Assays

A set of 12 microsatellite markers, previously proven to be highly performant for genetic olive characterization, were used (Table S7) [54,55,56]. PCR reactions were conducted in a final volume of 12.5 µL, according to di Rienzo et al. [6]. In brief, 1.25 µL of 10X Dream Taq Buffer, 0.6 µL of 2M dNTP, 1.25 µL of a mix of primers (2.5 µM), 0.2 µL of Dream Taq, and 7.7 µL H_2_O were added in each well containing 50 ng of DNA. PCR amplifications were performed in a C1000TM Thermal Cycler (Bio-Rad, Hercules, CA, USA), and the products were checked in 1.5% agarose gel. PCR products were detected by the automatic capillary sequencer ABI PRISM 3100 Avant Genetic Analyzer (Applied Biosystems, Foster City, CA, USA) with the internal molecular weight standard GeneScan Liz 600 dye (Applied Biosystems, Foster City, CA, USA). GeneMapper genotyping software v.3.7 (Applied Biosystems, Foster City, CA, USA) was used in order to carry out the sample analyses.

### 4.4. Data Analysis

The estimation of the following genetic indices was achieved by using GenALEx software v.6.5 (http://biology-assets.anu.edu.au/GenAlEx) [57]: number of alleles (Na), effective number of alleles (Ne), the Shannon’s information index (I) [58], observed (Ho) and expected (He) heterozygosity, and the fixation index (F) [59]. GenALEx was also used to estimate the number of private alleles [60], the marker-based relatedness (LRM) to infer the degree of relatedness for pairs of individuals [61], and the probability of two randomly chosen individuals having the same genotype on a set of 12 markers (probability of identity, PI) [62]. It was also used to carry out principal coordinate analysis (PCoA) based on inter–individual relationships using Nei’s unbiased genetic distance pairwise population matrix. The molecular variance among and within populations was then assessed by analysis of molecular variance (AMOVA). The informativeness of the primers was assessed by calculating the polymorphic information content (PIC) [63] with Cervus 3.0 software [64], as well as to estimate the frequency of null alleles.

The genetic relationships between the 77 olive samples were also estimated by using the Ward’s hierarchical clustering method based on a dissimilarity matrix using DARWIN software v.6.0.010 (http://darwin.cirad.fr), with bootstrapping of 1000 replicates to determine the support for each node [65]. 

Population genetic structure was assessed by using the Bayesian model-based clustering analysis [66] implemented in STRUCTURE software 2.3.4 using the admixture model. To obtain the best number of subpopulations (K) for the olive collection, ten independent runs for each K (from 1 to 10) were performed, using 100,000 MCMC repetitions and 100,000 burn-in periods. Resulting data were analyzed by Structure Harvester software [67], which is based on the ad hoc statistic ∆K test [68]. Genotypes were assigned to defined populations if the value of the corresponding membership coefficient (qi) was higher than 0.6 [49], otherwise they were considered to be admixed. The pairwise Fst between groups defined by STRUCTURE analysis was also calculated using Genalex software [69].

## Figures and Tables

**Table 1:** The diversity indices of 12 simple sequence repeat (SSR) markers detected in 77 Tunisian olive genotypes: size range, number of alleles (*Na*), number of effective alleles (*Ne*), Shannon’s information index (*I*), heterozygosity observed (*Ho*) and expected (*He*), fixation index (*F*), polymorphism information content (PIC).

Locus	Size Range (bp)	*Na*	*Ne*	*I*	*Ho*	*He*	F	PIC
DCA03	231–255	10	5.89	1.91	0.92	0.83	−0.10	0.80
DCA05	194–212	7	3.04	1.44	0.77	0.67	−0.16	0.64
DCA09	162–206	13	5.94	2.09	0.85	0.83	−0.03	0.81
DCA15	246–270	4	1.84	0.88	0.39	0.45	0.14	0.42
DCA16	122–186	19	9.26	2.51	0.97	0.89	−0.09	0.88
DCA17	109–181	9	3.11	1.39	0.25	0.67	0.62	0.63
DCA18	165–191	10	4.76	1.76	0.61	0.79	0.21	0.76
GAPU71b	121–144	5	4.91	1.60	0.89	0.79	−0.12	0.76
GAPU101	170–218	9	6.75	1.96	0.97	0.85	−0.14	0.83
UDO28	115–169	17	6.77	2.20	0.79	0.85	0.07	0.83
UDO43	166–216	15	7.03	2.19	0.90	0.85	−0.06	0.84
EMOL	190–228	6	2.70	1.18	0.45	0.63	0.27	0.57
Total		124	62.00					
Mean		10.33	5.16	1.76	0.73	0.76	0.05	0.73

**Table 2:** Diversity indices *Na, Ne, Ho, He,* and *F,* obtained with 12 SSR markers in the four groups of Tunisian olive genotypes, based on the area of sampling.

Collections		*Na*	*Ne*	*Ho*	*He*	*F*
Reference (IO)	Total	92.0	55.7			
	Mean	6.4	4.21	0.67	0.70	0.078
Nurseries (GR)	Total	77.0	50.5			
	Mean	6.4	4.21	0.67	0.70	0.078
Ras Jbal (RJ)	Total	74.0	44.5			
	Mean	6.1	3.71	0.82	0.67	−0.219
Azmour (AZ)	Total	82.0	55.5			
	Mean	6.8	4.63	0.79	0.73	−0.066

## Supplementary Materials

The following are available online at https://www.mdpi.com/2223-7747/9/3/382/s1, Figure S1: Probability of identity for the 4 groups of olive genotypes considered in this study. A minimum of 3 microsatellite loci were needed to meet the PID threshold of P < 0.01 [62], Figure S2: Allelic patterns across the four groups considered in the study, based on geographic origin; Figure S3: a) Mean of estimation ln probabilistic data of Tunisian Olive samples; b) Graph of delta K values to determine the best number of populations present in olive germplasm collection. The best K was at K = 3. Table S1: SSR profiles of 77 Tunisian accessions. Alleles length are expressed in bp, Table S2: List of pairwise relatedness based on LRM estimator [61], Table S3: List of genotypes harboring private alleles at different SSR loci, Table S4: The partitioning of genetic variation within and among groups obtained with AMOVA analysis for the 4 groups of olive accessions, Reference, Azmour, Ras Jbal and Growers, based on the area of sampling, Table S5: Pairwise population F_ST_ values that indicate the genetic differentiation between the 3 subpopulations (SP) detected by STRUCTURE at K = 3, Table S6: List of olive accessions considered on this study, with sampling site, area of collection and prevalent use, Table S7: List of the 12 microsatellite markers (SSR) used for molecular characterization of olive accessions. For each SSR, the identification code (SSR ID), repeat motif, primer sequence, bibliographic reference and annealing temperature (Ta) are reported.
