# Erratum: Recovery, Assessment, and Molecular Characterization of Minor Olive Genotypes in Tunisia. *Plants* 2020, *9*, 382

**DOI:** 10.3390/plants9050597

**Published:** 2020-05-07

**Authors:** Plants Editorial Office

**Affiliations:** MDPI, St. Alban-Anlage 66, 4052 Basel, Switzerland; plants@mdpi.com

The Editorial Office of Plants wants to make the following correction to the paper by Saddoud Debbabi, O., et al. (2020) [1]:

On page 1, the fourth author’s name should be Mahdi Fendri instead of “Mahdi Fendri Fendri”.

The manuscript will be updated and the original will remain online on the article webpage.

We would like to apologize for any inconvenience caused.
