# Supplementary material for: Recovery, Assessment, and Molecular Characterization of Minor Olive Genotypes in Tunisia
Source: Plants (Basel). 2020 Mar 20;9(3):382. doi: 10.3390/plants9030382 (PMC7154912; doi:10.3390/plants9030382)
Supplement: Supplementary file 1 [file plants-09-00382-s001.zip › S1 table rev..pdf]

Table S1. SSR profiles of 77 Tunisian accessions. Alleles length are expressed in bp.

Accessions	Origin	DCA03		DCA05		DCA09		DCA15		DCA16		DCA17		DCA18		GAPU71b		GAPU101		UDO28		UDO43		EMOL	
NEB_JEMAL1	NURSERIES	239	253	202	206	162	186	246	246	124	160	117	117	181	185	123	144	190	200	137	147	176	216	190	190
CHEMLALI_ JERBA	NURSERIES	233	243	206	206	178	204	256	256	148	160	115	115	173	175	127	130	182	182	131	167	176	176	198	198
CHEMLALI_SFAX1	NURSERIES	233	239	200	200	174	174	246	256	122	144	115	115	175	179	123	144	182	218	149	149	168	216	198	198
CHETOUI1	NURSERIES	233	239	206	206	194	194	246	246	122	172	117	117	175	179	121	127	182	218	123	149	176	216	190	190
OUESLATI1	NURSERIES	243	243	202	206	178	194	246	246	122	172	117	117	175	175	127	130	182	218	149	149	176	176	198	198
SAYALI1	NURSERIES	239	239	200	202	174	194	246	256	122	122	115	115	179	185	123	144	182	218	149	149	176	216	228	228
CHEMCHALI_GAFSA1	NURSERIES	233	243	194	206	174	194	246	246	138	148	115	115	179	179	123	130	192	206	131	149	176	186	198	228
BIDH_HMAM1	NURSERIES	237	249	202	200	162	186	246	246	160	172	117	117	177	177	121	123	190	200	123	137	174	176	190	190
GERBOUI1	NURSERIES	233	239	206	206	194	202	246	246	122	158	117	117	175	177	130	130	182	218	123	147	176	216	190	198
RKHAMI1	NURSERIES	233	239	206	206	194	202	246	246	122	158	117	117	175	177	130	130	182	218	123	147	176	216	190	198
ZARRAZI_MABROUKA	NURSERIES	233	243	202	206	162	204	246	256	138	172	143	143	173	173	130	144	190	200	131	149	174	176	198	198
TAMRI_DOUIRET	NURSERIES	239	239	194	202	178	204	246	256	122	164	109	115	179	191	123	130	182	198	131	131	176	180	198	198
CHAIBI_ONTHA	NURSERIES	233	239	194	206	194	194	246	246	122	138	117	117	173	179	121	127	192	206	0	0	176	216	190	190
NDB	NURSERIES	243	249	206	206	162	174	246	246	148	176	117	117	173	177	144	144	200	206	147	149	174	216	190	198
ASCOLANA	NURSERIES	233	249	206	208	194	206	246	246	124	152	115	117	165	173	123	144	198	200	123	131	176	216	198	198
BELLA_DI_CERIGNOLA	NURSERIES	249	253	206	208	162	182	246	246	152	176	115	117	171	177	121	123	200	218	0	0	174	176	198	198
CORELEA	NURSERIES	243	253	194	206	162	194	246	246	124	152	117	117	179	179	121	130	192	218	131	147	174	176	198	198
FRANTOIO	NURSERIES	237	243	198	206	182	204	246	246	148	152	117	143	179	179	123	144	182	198	149	167	176	216	198	198
REGUEB	NURSERIES	233	239	200	208	174	206	246	246	122	152	115	115	165	173	123	144	192	200	123	123	208	216	198	198
HAOUARIA	NURSERIES	233	239	194	206	174	194	246	246	144	176	165	181	175	175	121	123	192	198	149	149	176	216	198	198
NEB	RAS_JBAL	243	249	206	208	172	194	246	266	126	148	115	115	177	177	127	144	190	190	141	147	172	174	192	198
UNKNOWN1	RAS_JBAL	243	249	206	208	172	194	246	266	126	148	115	115	177	177	127	144	190	218	141	147	172	174	192	198
MESKI2	RAS_JBAL	243	249	206	208	172	194	246	266	126	148	115	115	177	177	127	144	190	218	141	147	172	174	192	198
RAJOU1	RAS_JBAL	239	253	194	206	194	204	246	246	122	172	115	143	179	179	121	130	206	218	123	131	210	214	192	198
NIBI1	RAS_JBAL	235	253	194	208	204	204	246	246	122	174	141	143	177	181	123	130	190	206	131	149	214	216	190	198
RAJOU2	RAS_JBAL	239	253	194	206	194	202	246	246	122	172	115	143	0	0	121	130	206	218	123	131	210	214	192	198
LIMI1	RAS_JBAL	239	245	206	208	186	194	246	246	122	174	115	115	177	177	127	130	190	218	131	131	174	214	190	198
OCTOUBRI	RAS_JBAL	239	253	194	206	194	206	246	246	122	172	115	143	177	179	121	130	206	218	123	131	210	212	192	198
CHAMI	RAS_JBAL	0	0	206	208	172	194	246	246	126	148	115	115	177	177	127	144	190	218	141	147	172	174	192	198
NIB2	RAS_JBAL	243	249	206	208	172	194	246	266	126	148	115	115	177	177	127	144	190	218	141	147	172	174	192	198
RAJOU3	RAS_JBAL	239	249	194	206	194	206	246	246	122	172	115	143	177	179	121	130	206	218	123	131	210	214	192	198
UNKNOWN2	RAS_JBAL	231	239	206	212	184	194	246	266	146	174	115	127	175	175	123	127	192	206	149	165	166	174	192	192
UNKNOWN3	RAS_JBAL	231	239	198	206	184	194	246	246	122	172	0	0	171	179	127	127	190	206	139	149	166	214	192	192
BESBESSI2	RAS_JBAL	243	249	206	208	172	194	246	266	126	148	115	115	177	177	127	144	190	218	141	147	172	174	192	198
UNKNOWN4	RAS_JBAL	243	249	206	208	172	194	246	266	126	148	115	115	177	177	127	144	190	218	141	147	172	174	196	198
UNKNOWN5	RAS_JBAL	231	249	202	206	186	194	246	270	162	176	115	115	177	185	123	144	190	218	137	147	176	208	196	198
UNKNOWN6	RAS_JBAL	231	249	202	206	186	194	246	270	162	176	115	115	177	185	123	144	190	218	137	147	176	208	192	198
UNKNOWN7	RAS_JBAL	249	253	206	206	162	194	246	266	126	176	115	115	177	187	121	144	200	218	123	147	172	210	192	192
UNKNOWN8	AZMOUR	231	243	206	206	162	186	246	270	148	154	115	115	181	181	121	123	190	200	139	149	174	214	192	192
LIMI2	AZMOUR	239	243	206	206	172	194	246	246	158	172	115	115	179	185	121	121	198	206	121	121	174	212	192	192
SAYALI2	AZMOUR	237	249	202	206	162	186	246	270	162	172	115	115	177	177	121	123	190	200	123	137	172	174	190	190
UNKNOWN9	AZMOUR	231	243	200	206	162	194	246	256	162	172	113	115	173	181	121	123	170	192	123	157	174	214	190	198
BESBESSI3	AZMOUR	243	249	206	208	172	194	246	266	126	148	115	115	177	177	127	144	190	218	141	147	172	174	192	198
NEB_JEMAL2	AZMOUR	231	255	194	206	186	194	246	246	144	176	0	0	173	175	121	123	192	218	123	157	174	174	190	190
RKHAMI2	AZMOUR	231	253	194	206	172	184	246	266	124	146	113	115	173	179	123	130	192	218	123	149	166	210	190	190
CHETOUI_REF	AZMOUR	231	239	194	206	194	194	246	246	122	174	115	115	175	179	121	127	192	206	123	149	174	214	192	192
CHEMLALI_AZMOUR	AZMOUR	231	239	194	200	172	172	266	266	122	146	113	113	175	179	123	144	192	198	149	167	166	212	198	198
UNKNOWN10	AZMOUR	239	239	206	212	172	194	246	266	122	186	0	0	179	181	121	127	190	206	149	165	174	184	192	192
CHEMLALI	AZMOUR	231	239	206	212	184	194	246	266	146	174	115	127	175	177	123	127	192	206	149	165	166	174	192	192
UNKNOWN11	AZMOUR	231	243	200	206	162	196	246	256	162	172	113	115	173	181	121	123	190	206	131	169	174	214	190	198
OCTOUBRI2	AZMOUR	245	253	194	206	162	186	246	266	148	172	115	115	173	177	121	144	200	206	145	161	172	214	198	198
ZALMATI	IO	231	239	194	200	172	172	266	266	124	146	113	113	175	179	123	144	192	198	0	0	166	212	198	198
GERBOUI2	IO	245	249	194	208	194	204	246	246	154	174	113	113	171	177	121	127	190	200	149	149	174	214	190	198
CHEMLALI_ONTHA	IO	231	243	194	198	172	176	256	266	146	162	113	113	173	179	130	144	192	198	149	167	166	166	198	204
NEB_JEMAL_TATAOUINE	IO	233	243	200	206	172	184	246	270	160	174	141	143	175	177	123	144	182	200	147	167	170	172	190	190
OUESLATI2	IO	243	243	202	206	176	194	246	270	122	174	115	115	175	175	127	130	192	200	149	149	174	174	196	198
CHEMLALI_JERBA	IO	231	239	194	200	172	172	266	266	122	146	113	113	175	179	123	144	192	198	137	137	166	212	198	198
ZARRAZI_ZARZIS	IO	243	249	206	206	162	172	246	246	148	174	115	143	173	177	144	144	200	206	119	119	172	212	190	198
SAYALI3	IO	231	239	194	206	194	194	246	246	124	178	115	115	175	179	121	127	192	206	115	127	174	214	192	192
CHEMLALI_ZARZIS	IO	239	239	198	206	172	184	246	256	148	148	113	113	175	179	127	144	190	206	131	169	186	212	198	198
RKHAMI3	IO	231	243	202	206	172	186	246	246	148	172	113	113	175	179	123	130	192	200	123	149	178	210	198	198
CHETOUI2	IO	231	239	194	206	194	194	246	246	122	172	115	115	175	179	121	127	192	206	123	149	174	214	192	192
BIDH_HMAM2	IO	243	249	194	206	162	204	246	246	148	172	113	115	177	179	121	130	200	206	123	131	174	210	198	198
CHEMCHALI_GAFSA2	IO	231	243	194	206	172	194	246	270	122	148	113	113	179	179	123	130	192	206	131	149	174	184	192	198
CHEMLALI_SFAX2	IO	231	239	194	200	172	172	266	266	124	146	113	113	175	179	123	144	192	198	149	167	166	212	198	198
CHEMLALI_TATAOUINE	IO	231	243	194	206	176	206	256	266	148	162	113	113	173	173	127	130	190	198	131	167	174	174	198	198
BAROUNI	IO	243	249	206	208	174	194	246	246	124	148	117	117	177	177	127	144	182	218	141	147	174	176	190	198
MESKI1	IO	239	245	206	208	186	194	246	246	122	170	117	117	177	177	127	130	182	198	131	131	176	216	190	198
TOFFAHI	IO	239	243	206	206	174	204	246	246	122	148	115	115	173	179	130	144	198	206	131	149	176	216	198	198
TOUNSI	IO	233	249	206	206	162	194	246	246	148	172	115	117	173	179	123	144	200	206	149	149	176	216	198	198
MARSALINE	IO	243	253	206	206	162	174	246	256	148	170	117	117	179	185	121	130	182	214	147	149	208	216	190	198
JEMRI_BOUCHOUKA	IO	233	243	206	206	174	194	246	246	122	148	115	115	179	179	123	130	182	218	0	0	176	186	190	198
FAKHARI	IO	243	249	206	206	162	178	246	246	138	148	117	117	173	177	144	144	200	206	147	149	174	216	190	198
BELDI	IO	243	249	206	206	162	174	246	246	148	172	117	117	171	177	144	144	200	206	147	149	174	216	190	198
BESBESSI1	IO	243	249	206	208	174	194	246	246	124	148	117	117	177	177	127	144	190	218	141	147	174	176	190	198
JEMRI_BC	IO	239	245	206	208	186	194	246	246	148	160	117	117	177	177	127	130	190	218	0	0	176	216	190	198
CHEMLALI_TATAOUINE	IO	233	243	206	206	178	204	256	256	138	148	115	115	173	173	127	130	190	198	131	131	176	176	198	198
