# Supplementary material for: Recovery, Assessment, and Molecular Characterization of Minor Olive Genotypes in Tunisia
Source: Plants (Basel). 2020 Mar 20;9(3):382. doi: 10.3390/plants9030382 (PMC7154912; doi:10.3390/plants9030382)
Supplement: Supplementary file 1 [file plants-09-00382-s001.zip › S2 table rev..pdf]

Table S2: List of pairwise relatedness based on LRM estimator (Lynch and Ritland, 1999).

**LRM = 0.500**	
GERBOUI1_GR	RKHAMI1_GR
UNKNOWN1_RJ	MESKI2_RJ
UNKNOWN1_RJ	NIB2_RJ
MESKI2_RJ	NIB2_RJ
UNKNOWN1_RJ	BESBESSI2_RJ
MESKI2_RJ	BESBESSI2_RJ
NIB2_RJ	BESBESSI2_RJ
UNKNOWN1_RJ	BESBESSI3_AZ
MESKI2_RJ	BESBESSI3_AZ
NIB2_RJ	BESBESSI3_AZ
BESBESSI2_RJ	BESBESSI3_AZ
ZALMATI_IO	CHEMLALI_SFAX2_IO
**0.500> LRM <0.374**	
UNKNOWN2_RJ	CHEMLALI_AZ
UNKNOWN1_RJ	CHAMI_RJ
MESKI2_RJ	CHAMI_RJ
CHAMI_RJ	NIB2_RJ
CHAMI_RJ	BESBESSI2_RJ
CHAMI_RJ	BESBESSI3_AZ
NEB_RJ	UNKNOWN1_RJ
NEB_RJ	MESKI2_RJ
NEB_RJ	NIB2_RJ
NEB_RJ	BESBESSI2_RJ
NEB_RJ	BESBESSI3_AZ
CHEMLALI_AZMOUR_AZ	CHEMLALI_SFAX2
CHEMLALI_AZMOUR_AZ	ZALMATI_IO
ZALMATI_IO	CHEMLALI_JERBA
BAROUNI_IO	BESBESSI1_IO
NEB_RJ	CHAMI_RJ
UNKNOWN5_RJ	UNKNOWN6_RJ
CHETOUI_REF_AZ	CHETOUI2_IO
OCTOUBRI_RJ	RAJOU3_RJ
UNKNOWN1_RJ	UNKNOWN4_RJ
MESKI2_RJ	UNKNOWN4_RJ
NIB2_RJ	UNKNOWN4_RJ
BESBESSI2_RJ	UNKNOWN4_RJ
UNKNOWN4_RJ	BESBESSI3_AZ
CHEMLALI_AZMOUR_AZ	CHEMLALI_JERBA_IO
RAJOU1_RJ	RAJOU2_RJ
CHAMI_RJ	UNKNOWN4_RJ
NEB_RJ	UNKNOWN4_RJ

Reference (IO); Nurseries (GR); Ras_Jbal (RJ); Azmour (AZ)
