# Supplementary material for: Recovery, Assessment, and Molecular Characterization of Minor Olive Genotypes in Tunisia
Source: Plants (Basel). 2020 Mar 20;9(3):382. doi: 10.3390/plants9030382 (PMC7154912; doi:10.3390/plants9030382)
Supplement: Supplementary file 1 [file plants-09-00382-s001.zip › S3 Table rev..pdf]

Supplementary Table 3: List of genotypes harboring private alleles at different SSR loci.

Collection (No. of accessions)	Genotype	locus	allele (bp)
NURSERIES (18)	CHEMLALI_SFAX1	UDO43	168
	TAMRI_DOUIRET	DCA17	109
		DCA18	191
		UDO43	180
		DCA16	164
	HAOUARIA	DCA17	165, 181
RAS_JBAL (18)	NIBI1	DCA03	235
	UNKNOWN7	DCA18	187
AZMOUR (13)	LIMI2	UDO28	121
	UNKNOWN9	GAPU101	170
	NEB_JEMAL2	DCA03	255
	UNKNOWN10	DCA16	186
	UNKNOWN11	DCA09	196
	OCTOUBRI2	UDO28	145, 161
REFERENCE (IO) (26)	CHEMLALI_ONTHA	EMOL	204
	NEB_JEMAL_TATAOUINE	UDO43	170
	ZARRAZI_ZARZIS	UDO28	119
	SAYALI3	DCA16	178
		UDO28	115, 127
	RKHAMI3	UDO43	178
	MARSALINE	GAPU101	214
