# Supplementary material for: Recovery, Assessment, and Molecular Characterization of Minor Olive Genotypes in Tunisia
Source: Plants (Basel). 2020 Mar 20;9(3):382. doi: 10.3390/plants9030382 (PMC7154912; doi:10.3390/plants9030382)
Supplement: Supplementary file 1 [file plants-09-00382-s001.zip › S4 table rev..pdf]

Table S4: The partitioning of genetic variation within and among groups obtained with AMOVA analysis for the 4 groups of olive accessions, Reference, Azmour, Ras Jbal and Growers, based on the area of sampling.

Source	df	SS	MS	Variance components	%
Among Pops	3	87.534	29.178	1.071***	11
Within Pops	73	654.479	8.965	8.960***	89
Total	76	742.013		10.036***	100

P-value is based on 1,000 permutations.
df: degree of freedom; SS: sum of squares; MS: mean squares; %: percentage of total variation.
