# Supplementary material for: Recovery, Assessment, and Molecular Characterization of Minor Olive Genotypes in Tunisia
Source: Plants (Basel). 2020 Mar 20;9(3):382. doi: 10.3390/plants9030382 (PMC7154912; doi:10.3390/plants9030382)
Supplement: Supplementary file 1 [file plants-09-00382-s001.zip › S5 table rev.pdf]

Supplementary Table S5: Pairwise population F_{ST} values that indicate the genetic differentiation between the 3 subpopulations (SP) detected by STRUCTURE at K = 3.

	**SP1**	**SP2**	**SP3**
**SP2**	0.206		
**SP3**	0.213	0.080	
