# Supplementary material for: Recovery, Assessment, and Molecular Characterization of Minor Olive Genotypes in Tunisia
Source: Plants (Basel). 2020 Mar 20;9(3):382. doi: 10.3390/plants9030382 (PMC7154912; doi:10.3390/plants9030382)
Supplement: Supplementary file 1 [file plants-09-00382-s001.zip › S6 Table rev.pdf]

Table S6. List of olive accessions considered on this study, with sampling site, area of collection and prevalent use.

Sampling site	Name	Origin	Use
Reference collection (Institut de l'Olivier) (26)	BAROUNI	North	Table
	BELDI	North	Oil and table
	BESBESSI1	North	Table
	BIDH_HMAM2	Center	Oil and table
	CHEMCHALI_GAFSA2	South	Oil and table
	CHEMLALI_JERBA_IO	South	Oil
	CHEMLALI_ONTHA	South	Oil
	CHEMLALI_SFAX2	Center	Oil
	CHEMLALI_TATAOUINE1	South	Oil
	CHEMLALI_TATAOUINE2	South	Oil
	CHEMLALI_ZARZIS	South	Oil
	CHETOUI2	North	Oil
	FAKHARI	South	Oil
	GERBOUI2	North	Oil and table
	JEMRI_BC	South	Oil and table
	JEMRI_BOUCHOUKA	South	Oil and table
	MARSALINE	North	Oil and table
	MESKI1	North	Table
	NEB_JEMAL_TATAOUINE	North	Oil and table
	OUESLATI2	Center	Oil
	RKHAMI3	North	Oil
	SAYALI3	North	Oil and table
	TOFFAHI	South	Oil and table
	TOUNSI	North	Table
	ZALMATI	South	Oil
	ZARRAZI_ZARZIS	South	Oil and table
Nurseries (20)	ASCOLANA	Italy	Table
	BELLA_DI_CERIGNOLA	Italy	Table
	BIDH_HMAM1	Center	Oil and table
	CHAIBI_ONTHA	South	Oil
	CHEMCHALI_GAFSA1	South	Oil and table
	CHEMLALI_ JERBA_GR	South	Oil
	CHEMLALI_SFAX1	Center	Oil
	CHETOUI1	North	Oil
	CAROLEA	Italy	Oil
	FRANTOIO	Italy	Oil
	GERBOUI1	North	Oil and table
	HAOUARIA	North	Oil
	NDB	Italy	Oil
	NEB_JEMAL1	South	Oil and table
	OUESLATI1	Center	Oil
	REGUEB	Center	Oil
	RKHAMI1	North	Oil
	SAYALI1	North	Oil
	TAMRI_DOUIRET	South	Oil
	ZARRAZI_MABROUKA	South	Oil and table
Ras_Jbal (18)	BESBESSI2	North	Oil
	CHAMI	North	Oil
	LIMI1	North	Oil
	MESKI2	North	Table
	NEB	North	Oil and table
	NIB2	North	Oil
	NIBI1	North	Oil
	OCTOUBRI	North	Oil
	RAJOU1	North	Oil
	RAJOU2	North	Oil
	RAJOU3	North	Oil
	UNKNOWN1	North	Oil
	UNKNOWN2	North	Oil
	UNKNOWN3	North	Oil
	UNKNOWN4	North	Oil
	UNKNOWN5	North	Oil
	UNKNOWN6	North	Oil
	UNKNOWN7	North	Oil
Azmour (13)	BESBESSI3	North	Table
	CHEMLALI	North	Oil
	CHEMLALI_AZMOUR	North	Oil
	CHETOUI_REF	North	Oil
	LIMI2	North	Oil
	NEB_JEMAL2	North	Oil and table
	OCTOUBRI2	North	Oil
	RKHAMI2	North	Oil
	SAYALI2	North	Oil

UNKNOWN10	North	Oil
UNKNOWN11	North	Oil
UNKNOWN8	North	Oil
UNKNOWN9	North	Oil

( ) Number of samples for each group
