# Supplementary material for: Recovery, Assessment, and Molecular Characterization of Minor Olive Genotypes in Tunisia
Source: Plants (Basel). 2020 Mar 20;9(3):382. doi: 10.3390/plants9030382 (PMC7154912; doi:10.3390/plants9030382)
Supplement: Supplementary file 1 [file plants-09-00382-s001.zip › S7 Table.pdf]

Table S7. List of the 12 microsatellite markers (SSR) used for molecular characterization of olive accessions. For each SSR, the identification code (SSR ID), repeat motif, primer sequence, bibliographic reference and annealing temperature (Ta) are reported.

SSR ID	Repeat motif	Primer sequence (5'-3')	Bibliographic reference	Ta
DCA03	$(GA)_{19}$	F 5'-CCCAAGCGGAGGTGTATATTGTTAC-3'<br>R 5'-TGCTTTTGTCGTGTTTGAGATGTTG-3'	Sefc et al. (2000)	50°C
DCA05	$(GA)_{15}$	F 5'-AACAAATCCCATACGAACTGCC-3'<br>R 5'-CGTGTTGCTGTGAAGAAAATCG-3'	Sefc et al. (2000)	50°C
DCA09	$(GA)_{23}$	F 5'-AATCAAAGTCTTCCTTCTCATTTCG-3'<br>R 5'-GATCCTTCCAAAAGTATAACCTCTC-3'	Sefc et al. (2000)	55°C
DCA15	$(CA)_3G(AC)_{14}$	F 5'-GATCTTGTCTGTATATCCACAC -3'<br>R 5'-TATACCTTTTCCATCTTGACGC -3'	Sefc et al. (2000)	50°C
DCA16	$(GT)_{13}(GA)_{29}$	F 5'-TTAGGTGGGATTCTGTAGATGGTTG -3'<br>R 5'-TTTTAGGTGAGTTCATAGAATTAGC-3'	Sefc et al. (2000)	50°C
DCA17	$(GT)_9(AT)_7$AGATA$(AGA)_{38}$	F 5'- GATCAAATTCTACCAAAAATATA -3<br>R 5'-TAATTTTTGGCACGTAGTATTGG -3'	Sefc et al. (2000)	50°C
DCA18	$(CA)_4$ CT$(CA)_3(GA)_{19}$	F 5'-AAGAAAGAAAAAGGCAGAATTAAGC-3<br>R 5'-GTTTTCGTCTCTCTACATAAGTGAC-3'	Sefc et al. (2000)	50°C
GAPU71b	GA$(AG)_6(AAG)_8$	F 5'-GATCAAAGGAAGAAGGGGATAAA-3'<br>R 5'-ACAACAAATCCGTACGCTTG-3'	Carriero et al. (2002)	57-60°C
GAPU101	$(GA)_8(G)_3(AG)_3$	F 5'-CATGAAAGGAGGGGGACATA -3'<br>R 5'-GGCACTTGTTGTGCAGATTG -3'	Carriero et al. (2002)	57-60°C
UDO28	$(CA)_{23}$ $(TA)_3$	F 5'-CTGCAGCTTCTGCCCATAC-3'<br>R 5'- GCAGATCATCATTTGGCACT-3'	Cipriani et al. (2002)	56°C
UDO43	$(GT)_{12}$	F 5'-TCGGCTTTACAACCCATTTC-3'<br>R 5'-TGCCAATTATGGGGCTAACT-3'	Cipriani et al. (2002)	57°C
EMOL	$(GA)_{12}$	F 5'-CTTTCCAATATGGGCTCTCG -3'<br>R 5'-ATGGCACTTTACGGGAAAAA -3'	De La Rosa et al. (2002)	50°C
