# Supplementary material for: Recovery, Assessment, and Molecular Characterization of Minor Olive Genotypes in Tunisia
Source: Plants (Basel). 2020 Mar 20;9(3):382. doi: 10.3390/plants9030382 (PMC7154912; doi:10.3390/plants9030382)
Supplement: Supplementary file 1 [file plants-09-00382-s001.zip › Supplementary Figures.pdf]

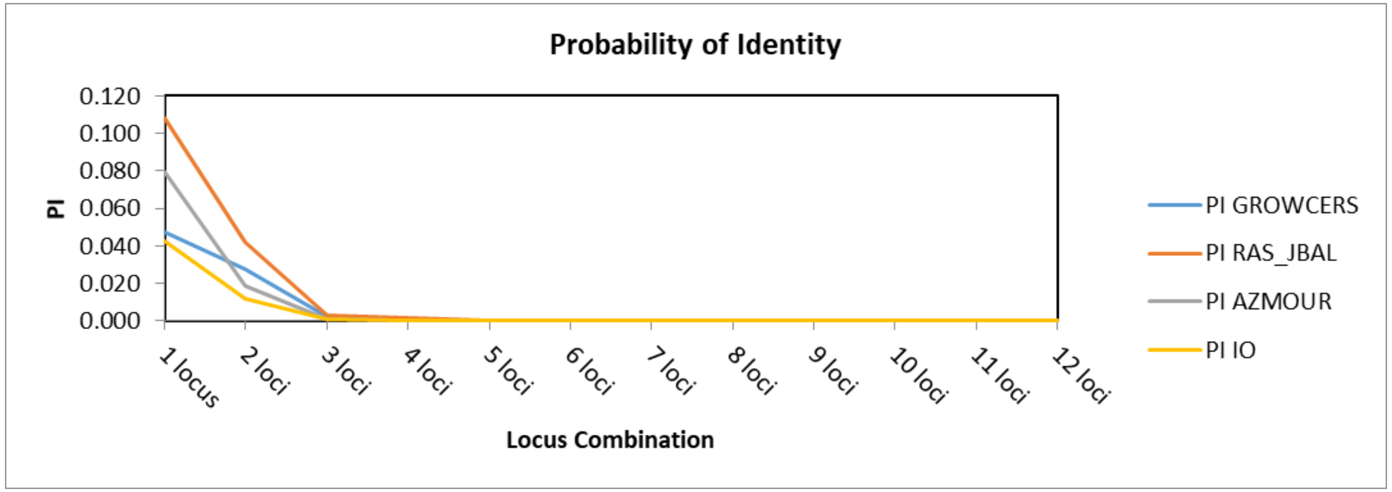

Supplementary Figure 1: Probability of identity for the 4 groups of olive genotypes considered in this study. A minimum of 3 microsatellite loci were needed to meet the PID threshold of $P < 0.01$ (Waits et al., 2001).

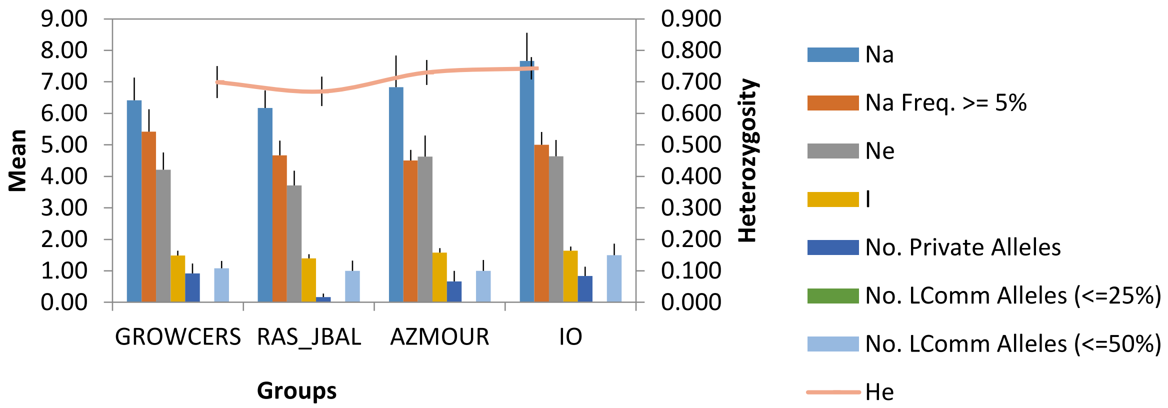

Supplementary Figure 2: Allelic patterns across the four groups considered in the study, based on geographic origin.

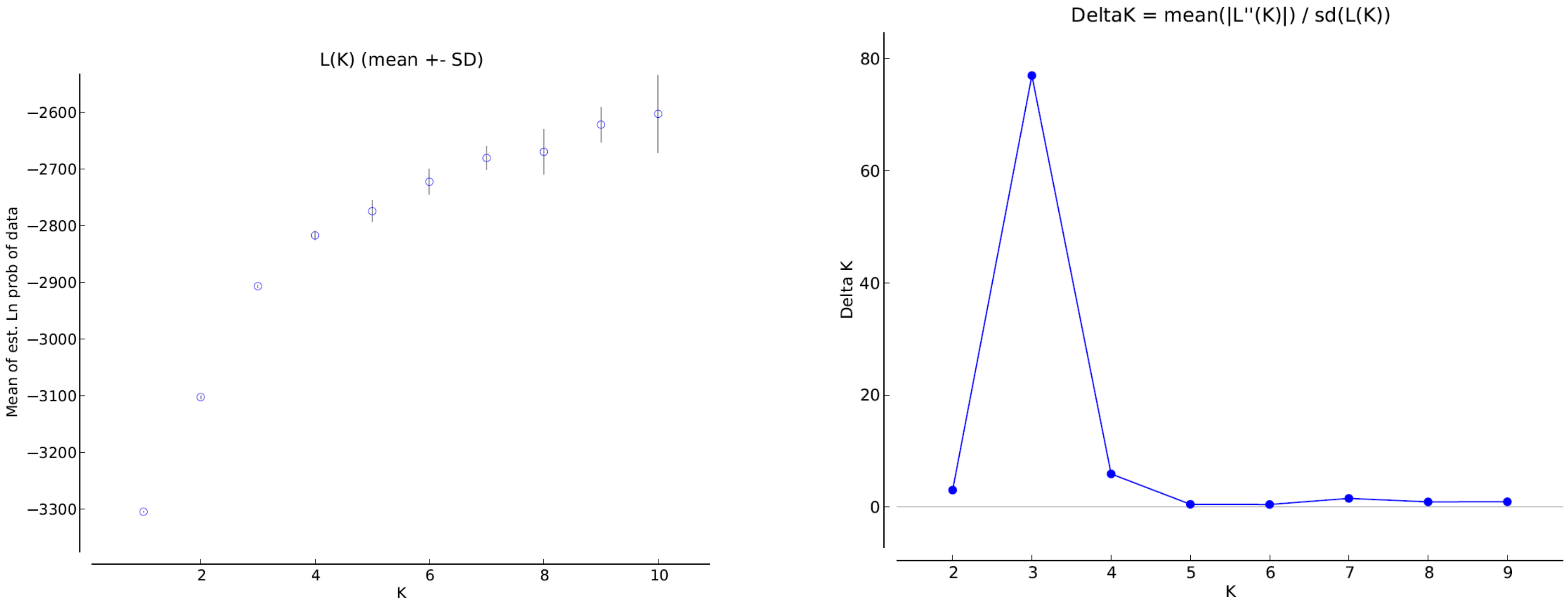

Supplementary Figure 3: a) Mean of estimation ln probabilistic data of Tunisian Olive samples; **b**) Graph of delta K values to determine the best number of populations present in olive germplasm collection. The best K was at K = 3.
